# Elements of the Etiology and Philosophy of Epidemics

**Published:** 1825-07-01

**Authors:** 


*Elements of the Etiology and Philosophy of Epidemics.* By JOSEPH MATHER SMITH, M.D. Fellow of the College of Physicians and Surgeons of the University of the State of New York, &c. 8vo. pp. 223. New York, 1824.

WE have often shewn that we belong to neither the contagionists nor non-contagionists—while we support the doctrine of *contingent* contagion, in the fevers of this, and even of tropical climates. The authentic documents which we published in the third number of this series, must, we think, convince the unprejudiced that, although a fever may commence epidemically, it may end contagiously, as was the case in the Bann sloop of war and other vessels.

Among the works which have issued from the contending parties, on both sides, we have seen none which have appeared more ingenious or philosophical than the one now before us. And although we shall have occasion to disagree with Dr. Smith, occasionally, in the course of our analysis, we give him full credit, not only for great talent and force of argument, but for candour and good faith—qualities which are not always to be found on either side of the question. We are happy, on another account, to pay our respects to this author—and that is, because he is an American. The petty bickerings and literary squibs which have too often shot across the Atlantic, eastward and westward, are now beginning to subside; and men of literature, as well as men of politics, are shaking hands across that vast ocean, which, we hope, will drown their animosities, while it binds the two nations in the harmonious ties of language, politics, commerce, literature, and science. In our own department (the medical) we trust we have not retarded but accelerated this desirable event; and we sincerely exhort our professional cotemporaries, on both sides of the Atlantic, to contribute, by example as well as precept, to this "consummation so devoutly to be wished."

In the work under review, "the author has attempted to arrange the causes of febrile and epidemic diseases, in systematic order; and to deduce from an examination of the nature and modus operandi of those causes, the laws which govern the rise, prevalence, and decline of epidemics; and also, the manner in which these diseases severally modify and supersede each other." He is far, however from presuming that he has completed this arduous task—"for he believes the period has not yet arrived, in which the various phenomena and laws of epidemics can be arranged with definite accuracy." His principal object therefore is to present, in part, the OUTLINE of a system of etiology, in which, he hopes, the leading facts relative to the prevalence of epidemic diseases will be exhibited, "in a method as conformable to nature as the present state of the science will admit." *Preface.*

In this disquisition, Dr. Smith has found (or fancied) himself under the necessity of introducing a considerable number of new terms,* which will give great offence to those who think that language, ideas, knowledge—are all fixed and irrevocable. Hear our author's apology for this innovation.

"The nomenclature of febrile diseases has long been enlarged to an extent that enables us to designate, by suitable terms, almost all their varieties; while that of their remote causes has been allowed to remain, for the most part, general, indeterminate and vague. As the successful prosecution of etiological inquiries depends, in a considerable measure, upon the precision with which one cause of disease can be discriminated

---

* We mean to the medical ear in this country; for our present author has the authority of Dr. Millar for most of the terms made use of in this work. *Ed.*

from another, it is hoped, that, however objectionable the terms he has introduced may appear to the fastidious philologist, they will be found more definite than the phraseology commonly employed in treating of the same subjects." 11.

Those indeed who are most *querulous* about the want of clear and definite terms to express our ideas respecting contagion, infection, &c. are most *clamorous* against any attempts to characterise them by accurate and appropriate definitions.

The first part of Dr. Smith's work consists of "an arrangement of the remote causes of febrile and epidemic diseases;" and of this attempt we shall endeavour to convey some idea to our readers.

Our author is aware of the difficulties attending etiological enquiries of this kind. Of the many remote causes of disease, we know nothing, except what is derived from observation of their *effects*—for instance, those epidemic influences which occasionally operate, with desolating fury, on whole communities. Hence the various and contradictory opinions which have prevailed among writers on these epidemics. No subject has excited more discussion than CONTAGION, in modern times. And yet we think these discussions are more creditable to the moderns, than the absence of such discussions or doubts among the boasted ancients, is to them. For nearly a thousand years, small pox, measles, and scarlet fever committed their ravages on the people, and were recorded by physicians, *who viewed them as varieties of the same disease, and having nothing to do with specific contagion!* Even Sydenham believed them to arise from atmospheric constitutions! So much for the lauded accuracy of the ancients. It was not till near the close of the 18th century, that the distinct character and independent origin of these three contagious disorders came to be universally perceived and acknowledged.* We agree with our author that little confidence is to be placed in the ancients, on the subject of contagion, after the glaring blindness which they evinced respecting the specific contagions now universally recognised.

"Among modern partisans, it has been the custom to cite Hippocrates, Celsus, Aretæus, and Trallian, to prove the noncontagiousness of fevers; and Thucydides, Lucretius, and Galen, to establish the opposite doctrine. These, it is acknowledged, are venerable authorities; but it is questionable, whether their observations and modes of philosophizing were sufficiently extensive and analytical, to enable them to decide a question which involves so many considerations as that relating to the contagiousness of fever. Their writings appear to furnish no

* See Bateman on Cutaneous Diseases.

facts calculated to elucidate the subject; yet if their opinions be admitted in the argument, those which favour the views of the anticontagionists must undoubtedly be regarded as the most worthy of confidence." 21.

After the middle of the 16th century, a contagious character was extended to every kind of fever, and even to consumption, influenza, and agues, at the very time when the exanthemata above mentioned, were considered to be unconnected with contagion! Now, as the same investigations which discovered the specific contagions of the exanthemata, raised doubts as to the communicability of fevers, our author infers that there must have been some good grounds for these same doubts. He (rather too sanguinely perhaps) prognosticates that, should enquiries go on with the same spirit as at present, "the dogma which asserts the specific contagiousness of yellow fever, *plague*, dysentery, typhus, and other fevers, will be forgotten, or only remembered as an error of the schools, before the lapse of the present century." On this we may remark that, with the exception of *plague*, there are few physicians, in this country at least, who believe that epidemic or endemic fevers *arise* from specific contagion; though facts daily teach us that these same fevers occasionally, and under particular circumstances, *give out* a something (call it contagion, malaria, infected atmosphere, diseased secretion, or what you please) which produces a similar disease in the healthy stander-by, who happens to come within its range. If we may venture a prognostic also, we would anticipate that this *will be*, as it assuredly *now is*, the more general opinion among *practitioners*. In all succeeding ages, however, we believe that *writers*, in their libraries, will be found to doubt every thing as well as to assert every thing, and thus amuse the world with their doubts and dogmas. When we see and know that nine-tenths of the really competent judges have come to the conclusions last mentioned, and when we see, every now and again, a writer launch forth from his heated brain the proud proclamation, that the whole world is of his opinion (all contagion, or no contagion*) we cannot but smile, and recollect the ostrich, who pushes his head into a bush and, as he can see nothing himself, concludes most logically that no creature can see him.

But setting aside, for the present, the question of contingent contagion, we are quite ready to admit, with Dr. Smith, (as we

* See the Proclamations of Sir Gilbert Blane on one side, and Dr. Maclean on the other.

said before) that the great epidemics and the local endemics arise from general causes, and not from specific contagion; but we are not so ready to agree with him in the following doctrine.

"Reasoning from these facts, and the well-known similarity of phenomena attending the prevalence of yellow fever and plague, we are led to conclude that the latter disease is also destitute of the quality of contagion, and that it is dependent upon a cause analogous to that of the former." 23.

In the first place, we must not decide such a question as this by *analogy*, but by the evidence of *facts* collected on the spot where plague prevails. If we examine such facts, collected or observed by those who have had personal communication with plague, we find that the great and preponderating weight of evidence is in favour of contagion. The analogies, then, of fireside travellers, such as Dr. Smith in America and Dr. Hancock in London, can only pass for what they are worth, which we believe to be just nothing at all. In the second place, we beg to ask Dr. Smith where he learnt the "well-known similarity of phenomena" attending plague and yellow fever? Is a disease which is almost as regularly characterised by a specific eruption as any of the exanthemata themselves, to be thus so readily identified with yellow fever, in which such a phenomenon is hardly ever seen?

From an extract, which we shall presently introduce, it will be evident that the difference between Dr. Smith and ourselves is rather in words than in things. He tells us that, so long ago as 1796, Dr. Richard Bayley, of New York, proposed that the term *contagion* should be restricted to morbid animal poisons, as those of small-pox, measles, &c.—and that the term *infection* should be limited to the "pestiferous effluvia arising from the excretions of the sick, and other species of filth." This distinction was soon approved of by several respectable writers, and Dr. Adams afterwards adopted it as his own, and without the least acknowledgement of the source whence he derived it.*

"Those who assert that contagion is the first essential of plague,

---

* *De mortuis nil nisi verum.* Various facts have come to our knowledge, which long ago convinced us that the late Dr. Adams was a man of extremely little and illiberal mind—prone to catch at anything that might appear original in him, without the sense of justice to acknowledge his obligations, *unless he feared detection*—which fear was his principal stimulus to candour. The editor of this journal once detected him in, and convicted him of, a wilful falsehood and plagiarism—which he (Dr. A.) never dared to rebut or deny.—*Ed.*

yellow fever, typhus, and dysentery, hold a doctrine which is consistent with itself; but those who admit that noxious exhalations originally produce those diseases, and, at the same time, contend that they are subsequently propagated by specific contagions, violate an established rule of philosophizing. They have recourse to more causes than are sufficient to explain the phenomena; and, what is equally unphilosophical, maintain, apparently without perceiving the absurdity, that two poisons, essentially different in their nature, are capable of producing the same specific disease. For example, they tell us that a miasmal poison, arising from the soil, will induce a peculiar form of fever; and that this fever will generate a contagious virus which is adequate to the production of the same disorder in a healthy person. This is obviously making like effects proceed from two very dissimilar causes; surely no one will alledge that marsh miasmata possess the qualities of poisons formed by animal secretion. The fact is, such poisons being different from miasmata, both in their origin and properties, must necessarily produce different diseases.

"The poisons of yellow fever and plague, like the exhalations which produce intermittent and remittent fevers, spring originally from the soil. In order, therefore, to render those fevers capable of communication, it is essentially requisite that the diseased body regenerate and multiply the same kinds of poisons. But it is plain that the constitution is no more adequate to this, than it is to the regeneration and subsequent exhalation of arsenic or corrosive sublimate when swallowed in doses sufficient to destroy life. The miasm, which is acting efficiently in a case of yellow fever or plague, never extends its influence beyond the patient; and its power is annihilated in the crisis of his disease. Hence we conclude, that all miasmal diseases are as destitute of contagious properties as the disorders occasioned by the mineral poisons.

"But here the interesting inquiry arises, whether individuals sick of a pestilential disease may always be approached with impunity? On this subject we have to remark, that although persons affected with yellow fever, or plague, are not to be avoided on account of any danger of contracting that peculiar form of disease by personal communication, *yet as vitiated human effluvia are highly deleterious, there is sometimes danger from this source, especially if there are many patients crowded into small and ill-ventilated apartments, if no attention is given to personal and domestic cleanliness, and if the disease be protracted to a stage in which the excretions of the body become putrescent and noisome.* It has been shown by Dr. Adams, that the poison, engendered under such circumstances, is not entitled to the name of contagion. It always, when it acts singly, *produces typhous fever, of greater or less severity,* whether it originates in the places occupied by those sick of plague, yellow fever, typhus, remittent fever, or by one confined with a broken leg. This we regard as a cardinal truth, which should be constantly kept in mind in our etiological researches. *In this manner typhus produces typhus;* and hence the erroneous conclusion that the disease is propagated by means of a specific contagion."—*Introduction,* 31.

The passages which we have marked in italics plainly prove that the whole of the objections to the doctrine of *contingent contagion* hinge on the interpretations given to certain words. Dr. Smith admits that, "in this manner, (crowding, want of ventilation, &c.) typhus produces typhus," which is all that we have ever contended for; but when he says that this *contingent* disease is *always typhus*, whatever was the original form of the fever, he says that, of which he offers no proof, and which we have amply shewn, by the fever in the Bann and at Ascension, to be *not conformable to experience*. Dr. Smith virtually contradicts his own doctrine, of typhus being *invariably* the result of this *infection*, arising in the crowded apartments of those who are sick of other fevers.

"As to the fevers occasioned by the exhalations of the soil, they can have, as before observed, no power of propagating disorders of their own nature; because those exhalations and vitiated human effluvia have different properties and produce different effects; *but should persons be exposed to the operation of both poisons at the same time, a new or modified form of disease will be the consequence.**

"From these observations, it will be perceived that, *we admit the facts recorded by practical writers, relative to the propagation of typhous diseases; but explain them differently from those who allege that they are specifically contagious.* Exceptions might be made to our reasoning, if it led us to view with indifference those means of checking the prevalence of typhus, which have been so successfully employed for that purpose in the cities of Great Britain. But so far is it from having that tendency, that it impels us to urge upon the medical police of every crowded city, the importance of instituting fever hospitals, as originally proposed and established by the distinguished Dr. Haygarth." 33.

We think we have now reduced the apparent discrepancy between Dr. Smith and ourselves to a mere *verbal* dispute, which, in reality, deserves no more than a slight explanation to set the question at rest between us. When we find our author, therefore, in the following pages, denying contagion, (contingent) we beg our readers may bear in mind that this denial is merely words, and not things.—But we must now come to our author's etiological arrangement.

ORDER 1.—*Contagion*—a poison generated by morbid animal secretion, possessing the power of inducing a like morbid action in healthy bodies, whereby it is re-produced, and indefinitely modified. This contagion can only be known by its

---

* How does this quadrate with the assertion that the new or contingent disease is *always* typhus?—*Rev.*

effects—and can only be divided into genera, by classifying the diseases which it produces. Dr. Hosack's classification is adopted, with a slight modification. GENUS 1. Contagion communicable exclusively by contact, its *species* being as follows:—*itch, syphilis, sibbens, launda of Africa, frambesia, elephantiasis, hydrophobia, vaccina.* These diseases cannot be conveyed through the medium of the air, but require actual *contact*—hence they are strictly *contagious,* in the etymological sense of the word. GENUS 2. Contagion communicable both by contact and by the atmosphere. These are liable to become *epidemic,* in contradistinction to those of the *first genus.* This genus contains, as *species,* the following:—*small-pox, measles, chicken-pox, scarlet fever, hooping cough.* To these, Dr. Hosack adds influenza and cynanche maligna. "But the *former*" (says our author, without proof,) "is *evidently* not contagious, and the *latter* is either a modification of scarlatina, or an atmospheric disease." One of the laws which govern these contagions is, that they are communicable in every season—in the heat of summer as well as in the cold of winter—in a pure, as well as in an impure air. Another law is, *general insusceptibility* to future attacks of the same disease, but with exceptions.

ORDER 2.—*Infection.* This is designated a febrific agent, "produced by the decomposition of *animal* and vegetable substances."*

"It usually exists in the state of a gas or *miasm,* and, in this form, occurs in filthy houses, ships, jails, hospitals, and cities; and also in marshes and fenny and low districts of country. Under the denominations of *marsh* or *paludal miasmata, exhalations of the soil, vegeto-animal effluvium, malaria, human effluvia, febrile* and *putrid contagion,* its various specific effects are detailed in the works of practical writers.

"The infectious miasms are considered as arising from decomposing animal and vegetable substances; first, because some of these always exist in the localities where they originate, and, secondly, because there is no evidence that any other species of matter are capable of producing them." 43.

* Our author has wisely avoided the error into which Dr. Bancroft has fallen on this point. That gentleman resolutely denies that *animal* matters can have any thing to do in the production of these febrific miasmata. But we have lately exhibited the most unequivocal proof of the contrary opinion, or rather fact, in the cases of fatal fever produced by opening a grave, in the Island of Lintin. Besides, as no chemical examination of these miasmata has ever taken place, and as animal matters are abundant in every situation where paludal effluvia exist, where is the proof that animal decomposition has no share in the production of the said miasmata?

After commenting on Dr. Ferguson's opinions respecting the production of febrific miasmata, our author goes on to say :—

"But with respect to dead animal and vegetable substances, we have incontestable evidence that they emit effluvia which produce fevers. Now, there is, perhaps, no variety of soil on the earth, except in arid sandy deserts, and the higher polar regions, which does not contain more or less putrescent organic remains. *When we advert to the immense number and variety of insects, worms, and reptiles, and the animals of a higher order which are constantly perishing and mingling their elements with the mineral constituents of the soil;* and when we observe like phenomena occurring in the vegetable creation, the largest trees, and all the inferior plants sinking into the same common grave, we cannot avoid the belief, that it is to such materials we must look for the origin of miasmal exhalations.

"The smaller animals, and the vegetables which most readily undergo disorganization, are found in the greatest abundance in flat and marshy countries; and this appears to be one reason why such places are so prolific of unwholesome exhalations." 46.

In general, the miasmal poisons possess no sensible qualities of smell, taste, &c. There may be bad smells *accompanying* febrific miasmata, but whether these have any immediate connexion with the poisons themselves, we have no means of ascertaining. In fact, the existence of miasmata in the atmosphere has never yet been proved by chemistry or the senses. Many of the circumstances, however, in which they originate, are readily distinguished.

"The late Dr. Edward Miller, of New-York, in his 'Attempt to deduce a Nomenclature of certain Febrile and Pestilential Diseases from the nature and origin of their remote causes,' published in 1804, divided the miasmal poisons into two species; the first, comprising the exhalations of the soil, and the second, the effluvia generated by personal and domestic filth. These noxious principles, he says, 'must be considered as gaseous fluids floating on the surfaces, or surrounding the bodies, from which they are respectively exhaled; and hence, like the ethereal fluids of magnetism and electricity, they may properly be called *miasmatic atmospheres.*'

"'In order to distinguish these two *miasmatic atmospheres*,' he observes, 'and, at the same time, to duly fix in the mind the impression of the origin and production of them, it is judged expedient to designate each by terms which will invariably express the process of nature in their formation. As the Greek language has been generally resorted to in the framing of scientific nomenclature, I shall employ the adjective ΚΟΙΝΟΣ, *common* or *public*, to denote one species of miasma, and ΙΔΙΟΣ, *personal* or *private*, to denote the other. The application of these terms will be readily understood. That portion of air charged with miasmata, exhaled by solar heat from the surface of swampy grounds, or from masses of filth overspreading the open area of cities, according to this distinction is denominated *Atmosphera koino-miasmatica.* And that other small portion of air, contaminated by miasmata emitted from and surrounding the body, clothes, bedding, and furniture of persons immersed in the filth of their own excretions, and of those associated in the same family with them, accumulated, long retained, and acted upon by animal heat, is denominated *Atmosphera idio-miasmatica!*" " 49.

We think that, for the sake of precision in language, and to prevent so much verbal squabbling, an arrangement of the above kind would be very useful in the etiology of fevers. Doubtless some of our witty critical brethren will laugh at these KOINOS, and ridicule them as mere " word-*coining*;" but this is hardly fair, when attempts are made to define, with more precision, the vague ideas and terms scattered through medical writings and colloquial language.

GENUS I.—*Koino-miasma.* This genus comprehends the effluvia exhaled from the public filth of cities, and from the soil of marshes and campaign countries. It, also, properly includes the noxious emanations from animal and vegetable substances, accumulated and allowed to putrefy in cellars, storehouses, and the holds of ships. A high range of temperature, and the immersion of the corrupting materials in a certain degree of moisture, are generally essential to the production of this kind of miasma. It is diffusible through the common atmosphere, and, when widely extended, occasions a general prevalence of disease—or, in other words, an *epidemic.* In this genus our author arranges the miasms which produce plague, yellow fever, and the bilious remittent fevers—" diseases, for the most part, peculiar to tropical climates, and to the warm seasons of the temperate latitudes."

GENUS II.—*Idio-miasma.* This miasm is produced from the matter of perspiration, and the other excretions of the human body, accumulated in small and unventilated places, and acted on by heat.

" It occurs most frequently in the houses of the poor, and the apartments of the sick, and especially of those labouring under the typhous state of fever. It is commonly distinguished in the books by the terms vitiated human effluvia, typhous and putrid contagion.

" This poison is the source of genuine typhus, such as jail, hospital, and ship fevers. None of these forms of disease can be said to occur epidemically; for the miasm becomes innoxious when diffused in the atmosphere a few feet beyond the apartments in which it is engendered. It is extremely rare that the general atmosphere of a city becomes pestiferous from human effluvia.

"The fevers, produced by *Idio miasma*, are also distinguished from those arising from Koino miasma, by their appearing generally in the middle and higher latitudes; and, for the most part, in the colder seasons of the year; that is, during the period that houses are generally crowded, and not freely ventilated, owing to the inclemencies of the weather." 52.

The preceding genera, our author observes, comprise all the infectious sources of fever. But he remarks that they do not provide for "that combination of the two miasmal poisons, which produces compound fevers." Dr. Miller, in his classification, seemed to be aware of this defect, and thus observes —"it would be a subject of curious and interesting inquiry, how far these different febrile poisons are susceptible of being blended, and thereby of producing effects of a mixed kind; and likewise, how far the idio-miasmatic atmosphere, by means of high solar heat and other concurring circumstances, is capable of conversion into the koino-miasmatic atmosphere."*

Such a *conversion*, Dr. Smith regards as impossible, averring that there are no circumstances which can enable simple human effluvia to produce any other forms of fever than the species of genuine typhus. Of this we are by no means certain. Our author, however, allows that the miasm of typhus, when it unites with the exhalation from marshes, or the public filth of cities, may produce a disease of *a peculiar or mixed character*. "This compound source of disease, therefore, is highly interesting, both in a practical and scientific point of view, and is, we think, sufficiently distinct and well charactesed to be ranked as a genus." To this, therefore, we proceed.

Genus III.—*Idio-koino-miasma*. This genus, according to Dr. Smith, may be defined the combination of human effluvia with the exhalations of the soil; or, in other words, "the intimate union of the species of the first genus of *infection* with those of the second, producing, virtually, a *tertium quid*." But, as this source of disease has never received that particular consideration which our author thinks it deserves, we shall allow Dr. Smith to use his own words on the occasion.

"And, first, let us suppose the circumstances in which typhus originates to occur in summer, such as the crowding of individuals into small apartments, badly ventilated, and rendered offensive by personal and domestic filth. These causes would obviously produce typhus in its ordinary form. But suppose there exist at the same time those ex-

* Miller's Medical Works, p. 196.

halations which occasion plague and yellow fever, or intermittent and remittent fevers. Under such circumstances, we should not expect to see any one of those diseases fully and distinctly formed, but a disease of a novel or modified character.

"In this country, several memorable instances have occurred within a few years, which will enable us to exemplify the peculiar agency of *Idio-koino miasma.*

"The fever which prevailed in Bancker-Street, New-York, in the summer and autumn of 1820; and the cases of fever admitted into the Philadelphia Alms-House in the same season, were diseases arising from this cause.

"The diversity of opinion, which prevailed among gentlemen equally distinguished for their medical learning and experience, relative to the Bancker-Street fever, evinced that there was something novel in its character, or rather that it was different from the ordinary endemic diseases of our cities. By some it was unequivocally called *yellow fever*, while others with equal confidence pronounced it *typhus.* Of the former opinion were the Committee appointed by the Medical Society to inquire into the causes and character of the disease. The Committee produced abundant evidence that there existed in Bancker-Street and its vicinity, all the circumstances necessary to produce typhus. They carefully noted the extraordinary number of individuals residing in that district, their poverty, filthiness, and intemperance; and also the foul condition of the streets, courts, and alleys.

"In deducing their conclusions, it appears they proceeded on the assumption, that the causes of typhus are never operative but in winter; and consequently that the fever of Bancker-Street had nothing of the typhous character, but being malignant, and occurring in summer, could be no other than the pestilential *yellow fever.* It is here we think they erred; on the other hand, those who contended that the disorder was *typhus,* apparently forgetting that *koino miasma* was acting at the time, and which, conjoined with the influence of a high atmospheric temperature, was producing bilious remittent fevers in other parts of the city, were betrayed into an opposite error.

"Upon a careful review of all the facts connected with this subject, we think we are warranted in concluding, that the fever of Bancker-Street was neither genuine typhus nor bilious remittent, or yellow fever; but a distinct and compound fever, illustrative of what Dr. Miller considered the "interesting inquiry how far the different febrile poisons are susceptible of being blended, and thereby of producing disease of a mixed kind." 56.

Our author remarks, that this peculiar form of fever, *both* in New York and in Philadelphia, was, under similar circumstances, more prevalent and fatal among blacks than whites—so much so, that it obtained the popular name of the Negro fever. We agree with our intelligent author in the following passage, which cannot be too rigidly inforced on the minds of tropical practitioners, and the medical officers of our fleets and armies.

"Both koino miasma and idio miasma are frequently engendered on board of ships. 'Vessels abounding in animal and vegetable filth, and navigating the warm latitudes, on arriving in port during a hot season, will be apt to generate the former species of miasm, while such as sail on long voyages, and are crowded with passengers, who neglect, or are deprived of the means of cleanliness and ventilation, will be chiefly liable to produce the latter.'* The simultaneous generation, and consequent union of these poisons on ship-board, will induce a disease similar to that of Bancker-Street. Several interesting examples of this kind might be adduced. The same form of disease has been observed to occur in persons who have been exposed to koino miasma on shore, and who are subsequently embarked in crowded ships. Pringle tells us, that 'in the autumn of 1757, when our troops returned from the expedition to the Rade de Basque, several of the soldiers were brought into the hospital at Portsmouth, ill of a disorder, *compounded with the bilious and jail fever*. For when those men, upon being seized with the common fever of the season, were confined to the holds of the crowded transports, their distempers assumed a malignant form.' "† 59.

The plague of Athens, as recorded by Thucydides, appears to our author to be one of these hybrid or compound fevers, resulting from a combination of human and terrestrial miasmata.

"We are told that during the invasion of the Athenian territories by the Peloponnesians and their Allies, the people of Attica took refuge within the walls of the Capital; that the city was unable to receive so large a conflux of people; that many were forced to lodge in the turrets of the walls; and that a great part of the Priæus was portioned out to them for little dwellings. 'Besides this reigning calamity,' says Thucydides, 'the general removal from the country into the city was a heavy grievance, more particularly to those who had been necessitated to come thither; for as they had no houses, but dwelt all the summer season in booths, where there was scarce room to breathe, the pestilence destroyed with the utmost disorder, so that they lay together in heaps, the dying upon the dead, and the dead upon the dying. Some were tumbling over others in the public streets, or lay expiring round about every fountain, whither they had crept to allay their immoderate thirst.'‡ 'So urgent was the necessity,' to use the language of Dr. E. H. Smith, 'that contrary to the express prohibition, as it was supposed, of the Pythian Oracle, they seized upon the interdicted ground of the Pelasgic, and erected there their miserable huts. Set up wherever space was found, in the utmost disorder, and pressed together, they experienced no free circulation of the air, while their diminutive size provoked the sallies of a sarcastic poet, (Aristophanes) who compares them to buts or casks.'§

---

* Miller.
† Observations on the Diseases of the Army, p. 130.
‡ Smith's Translation, vol. i. p. 166.
§ New-York Med. Repository, vol. i. p. 15.

The population of the city at this time, is supposed to have been augmented from fifty thousand to more than four hundred thousand.

"When all these circumstances are duly considered, there cannot remain a doubt that vitiated human effluvia, and noxious exhalations from public filth, in a word that *Idio-koino miasma* was generated and rendered in a high degree malignant." 61.

That this was the cause which threatened to depopulate Athens is rendered probable, Dr. Smith naturally infers, from the nature and character of the disorder, which appears to have differed materially, if not essentially, from the modern plague of the Levant, inasmuch as buboes and carbuncles are not included in the symptoms of the Athenian scourge. It is true that in very rapidly fatal cases of the Turkish plague, the above symptoms do not always appear :—but, as many of the Athenians died on the seventh and ninth days of the disease, and even later, there was time for the occurrence of buboes ; and such a remarkable symptom was not likely to have escaped the observation of the accurate historian. Dr. Smith, therefore, comes to the conclusion, that the Athenian epidemic was not genuine plague, " but a malignant *idio-koino miasmal fever.*"

We now come to that part of the work which treats of the particular species of infection or of febrile miasmata.—Dr. Miller has remarked, that miasmata may be properly divided into the *mild* and *malignant*. Our author follows this division, but prefers giving them specific names, on the plan of modern chemists :—thus to the *mild* miasmal poisons he prefixes *proto* the Greek ordinal numeral—while to the *malignant* miasmata he prefixes the intensive particle *per*. The first kind, or rather grade, then, will stand *protokoino miasma*, while the second, or malignant, will be, of course, *perkoino miasma*. In this manner the species of idio-miasma, and idio-koino miasma will also be distinguished. For our own parts we attach no other importance to these distinctive appellations than we would to similar terms in nosology or chemistry. They facilitate, we imagine, the study of the particular subjects.

*Species* 1. *Protokoino Miasma*. This consists of the exhalations from the soil, which, in summer and autumn, produce intermittent and remittent fevers, and also derangement of the intestinal and hepatic functions. They are usually denominated marsh miasmata. That this species is different from that which produces yellow fever and plague cannot, our author thinks, be doubted—" for were they the same, we should find those diseases as frequently and extensively prevalent as intermittent and remittent fevers."

"*Protokoino miasma* is generated in situations where there is putrescent matter immersed in certain degrees of moisture, and acted upon by solar heat. The localities which furnish it in the greatest abundance are filthy cities, marshes, and flat countries. In wet seasons, swampy and low grounds are comparatively unproductive of the miasm, in consequence of being saturated, or inundated with water. In such seasons the dryer and more elevated tracts of country, which are usually healthy, are particularly liable to visitations of miasmal diseases. 'Dr. Dazille, in his treatise upon the diseases of the negroes in the West Indies, informs us,' says Dr. Rush, 'that the rainy season is the most healthy at Cayenne, owing to the neighbouring morasses being deeply overflcwed; whereas at St. Domingo a dry season is most productive of diseases, owing to its favouring those degrees of moisture which produce morbid exhalations. These facts will explain the reason why in certain seasons, places which are naturally healthy in our country become sickly, while those places which are naturally sickly escape the prevailing epidemic.'* Dr. Ferguson, in his interesting paper on the marsh poison, corroborates the statement of Dr. Dazille. In performing the duty assigned him of making a medico-topographical survey of the West India Colonies, he observed 'that the same rains which made a deep marshy country perfectly healthy, by deluging a well-cleared one, where there was any considerable depth of soil, speedily converted it, under the drying process of a vertical sun, into a hotbed of pestiferous miasmata.' "† 71.

A temperature below 35 or 40 of fahrenheit, and a state of perfect dryness or extreme humidity, may be considered incompatible with its generation. It appears to arise most abundantly from grounds which are undergoing the process of exsiccation, or which are occasionally irrigated, or moistened by showers, and at the same time steadily acted on by summer heat. Accordingly the margins of rivers, ponds, lakes, and certain points of savannas and swamps, are justly deemed more fruitful of miasmata than stagnant waters, or very hard or dry soils.

"In cities, the quantity of moisture and filth varies in different places; and consequently it must often happen that some spots will be in a condition more favourable to the production of febrific effluvia than others. Hence it is, that ordinary autumnal fevers are frequently observed to prevail most in certain neighbourhoods. The miasm exhaled from such spots sometimes extends over a whole town, and occasions a general epidemic. In like manner, the effluvium from marshes spreads over a considerable space of the circumjacent country, and produces a corresponding prevalence of disease. Sometimes it reaches places somewhat elevated, though its extension in certain directions is generally arrested by mountains, high ridges of land, and dense and lofty forests." 72.

---

* Medical Inquiries and Observations, vol. iii. p. 108.

† Philadelphia Med. and Phys. Journal, No. 13.

The soil of woodlands is less productive of *protokoino miasma* than marshes, and open flat countries—but the clearing of such lands is frequently followed by fevers. Newly turned-up soil, in unhealthy countries, is always dangerous, as it exposes to the sun a large surface covered with putrescent vegetable matters.

"*Protokoino miasma* is supposed to be specifically heavier than common air; for it extends along the surface of the soil to a considerable distance from its source, and is always most active in low situations. It appears to form a stratum, resting on the earth, which, like other vapours, may be agitated and dispersed by the winds. Its ascent in the atmosphere is varied by the rarefying influence of the solar heat. Thus, during the day it is elevated, but at night is condensed and descends with the dew. Hence the danger of sleeping on the earth, or in tents, and the greater salubrity of the upper stories of buildings. These observations will in part explain the fact, that, as the season advances, miasmal fevers multiply. The heats of summer, though more favourable to the production of the poison, are less so to its concentration, than the temperature of autumn." 74.

Our author thinks it questionable whether this measure can be conveyed in the form of *fomites*, from one place to another. We should think this is very unlikely to happen. We are disposed to agree in the following sentence, however, to wit:—"different countries, and different localities in the same country, probably furnish varieties of the species *protokoino miasma*." Of this, we think, there can be little doubt, if we may judge from their various effects. But although this poison may vary in its qualities, our author conceives that "the pathology of its diseases is every where the same." This, we think, is an assumption standing in need of further proof. From the following passage, it will be seen that our author differs from Dr. Bancroft, and several able modern writers, on the question of the yellow fever of the West Indies being only a grade of bilious remittent fever.

"In all countries, and especially in hot climates, the fevers occasioned by *Protokoino miasma* are sometimes attended with black vomit, and yellowness of the eyes and skin; circumstances which have led many observers to consider them of the same nature as the pestilential yellow fever, differing from it only in grade. This error has long been associated with doctrines otherwise true. In the West Indies, there is probably much difficulty in distinguishing the worst cases of *malignant* bilious fever from genuine yellow fever. To judge correctly, where there is any doubt relative to the nature of a febrile disease, several cases, occurring at the same time and place, should be attentively investigated. If a majority of these cases manifest the signs of bilious remittent fever, they should be regarded as distinct from yellow fever, notwithstanding the malignity of some of them; and on the other hand, if in the greater number, the characteristics of yellow fever clearly and unequivocally appear, they should be considered specifically different from *Protokoino miasmal* fevers.

"To this history of the milder *Species* of Koino miasma, we may add, that when viewed in its geographical relations, it is found to be indigenous in most countries of the torrid and temperate zones. During the summer and autumn, it is abundantly produced throughout the alluvial tract which stretches along the Atlantic sea-board from New-Jersey to the gulph of Mexico. It is also a prolific source of fever in the secondary region, west of the Alleghanies; and in the countries bordering on the great American lakes. In the north-eastern States, the formation of which is for the most part primitive, it is not so general; but in every place where there are swampy grounds, or the country is low and level, it frequently produces epidemics. Some of the more remarkable foreign sources of this poison are the fens of Lincolnshire, the low lands of Holland, and the Netherlands, the marshes of Italy, Hungary, the Indies, and the western shores of Africa. Indeed, next to the morbid influences of the atmosphere, it may be regarded as the most general cause of disease." 77.

*Species* 2. *Perkoino Miasma.* This species, according to our author, embraces the poisons of *yellow fever*, *plague*, the malignant effluvia of Batavia, and of some other parts of the East. We recommend the following extract to the serious attention of all reflecting men, whether contagionists or anti-contagionists. It appears to us to contain much sound reasoning, as well as solid facts.

"It is distinguished from the Species Protokoino miasma, by its more virulent and pestilential qualities, and by its occurring for the most part in cities. In crowded populations it affects great numbers at the same time, and usually prevails with destructive severity for a period of several months. Among commercial nations, its specific character is acknowledged in their prohibitory measures of quarantine.

"Although *Perkoino miasma* may constantly exist at some places within the tropics, yet its *epidemic* appearance, especially in temperate climates, is very irregular. It frequently prevails in the cities of neighbouring countries at the same time, or successively in different seasons. Sometimes, however, there are intervals of many years, during which it scarcely appears in the temperate latitudes.

"This poison, like the first species of koino miasma, is exhaled from masses of public filth, and soils containing putrescent matter; and is generated under a high range of temperature, and certain epidemic influences of the general atmosphere.

"In the United States and the Islands of the Antilles, where the yellow fever is most prevalent, it generally first appears in the lowest parts of sea-port towns; and particularly on the margins of rivers, and about docks constructed of perishable materials. Usually it commences its ravages in a small neighbourhood; and from this gradually extends in every direction, progressing uninterruptedly from street to street, and thus enlarging its circle, until arrested and destroyed by certain changes in the qualities of the atmosphere. Occasionally it appears in several parts of a city, in the same season; but then, always spreads in the manner just stated. Sometimes the grounds from which it emanates are not so narrowly circumscribed, but are of considerable extent, especially in tropical climates, and in sea-ports where public cleanliness is neglected.

"The source of the yellow fever poison, and the means by which the disease is spread through a city, are well known to be subjects fertile of controversy. In pursuing the history of *Perkoino miasma*, the questions in dispute will properly come under review.

"All admit, that in the cities of the middle latitudes, the poison makes its epidemic appearance in the localities above described; that its field of prevalence widens slowly and regularly from day to day; and that it spreads, not only through the lowest and filthiest, but occasionally to the higher and more cleanly streets.

"These facts have been urged to prove, that the poison of yellow fever is a specific *contagion*, introduced from a foreign source; and that the extension of the disease is owing to personal communication. This doctrine has but few advocates on this side of the Atlantic, and will no doubt soon be entirely exploded.

"The fact, however, is well established, that the agent which produces yellow fever is capable of being transported from one country to another; and there are strong reasons for believing that it is often engendered in ships while navigating the tropical seas. The frequent arrival of infected vessels from the West Indies, at the sea-port towns of the United States and of Spain, has given a colouring of truth to the hypothesis of exclusive importation. That the entrance of such vessels into port is dangerous, and often fatal to those who board or approach them, is at present a matter of universal notoriety. But a broad distinction should be made between this source of yellow fever, and that of a pestilential epidemic. Formerly, the anti-contagionists were over anxious to prove that the yellow fever which casually appeared in certain towns of the United States, originated from domestic causes; but the proofs of its importation, in several instances, are clear and indisputable. Thus the cases which occurred at the Wallabout in 1804, at Perth-Amboy in 1811, at Middletown, Connecticut, in 1819, and at the New-York Lazaretto in 1821, were satisfactorily traced to vessels recently arrived from the West Indies. It seems that the anti-contagionists feared that such concessions would invalidate the doctrine of the domestic origin of yellow fever; but this can never be the consequence of a fair investigation of the subject. The evidence in favour of the local production of *Perkoino miasma* is no less conclusive than that which establishes its introduction by ships. The following facts are deemed sufficient proofs of its local or domestic origin.

"1. The disease frequently appears in the first instance among those who have had no communication with infected ships, goods, or other articles. This fact is abundantly established by the testimony of many respectable American physicians.

"2. The disorder prevails as an *epidemic* only in the warm seasons of the year; that is, it commences in summer, and after raging for several months, disappears in the autumn; exhibiting in its course a rise, progress, and decline, like other epidemics produced by miasmal causes. It proceeds in the manner here stated, when there are no suspicious vessels at the wharves, or goods imbued with foreign infection in the town. Moreover, it never spreads beyond the atmosphere of a city, nor is it propagated by the sick when removed to the country, or to the healthy parts of a town—a decisive proof that its epidemic prevalence is not dependent upon a specific contagion.

"3. The early and entire desertion of an infected neighbourhood or district, and of a wide space around it, does not in the slightest degree tend to stay the extension of the poison, nor to diminish the danger of visiting the unihabited locality, so long as the weather continues favourable to the existence of the miasm, which is frequently for three or four months. In general there is much greater hazard in walking through the streets originally infected one or two months after the inhabi[illegible]s have removed, than at the commencement of an epidemic. These [illegible]cts are in no way reconcileable with the doctrine of contagion.

"But it may be asked, in what manner can it be satisfactorily determined, whether yellow fever originates from a foreign or domestic source, when there is a coincidence between the arrival of infected vessels, or the introduction of infected goods, and the commencement [illegible] epidemic?

"In some instances it may, on the first appearance of the disease, be difficult to answer this question; but, in the event, we think, it may be solved with accuracy. If the poison be imported, the disease will not prevail epidemically; it will affect those only who approach the infected vessels or their cargoes, and will totally disappear on removing them to a distance; whereas, if the disease be of domestic origin, it will continue to prevail through the season, whether the ships be removed or not—provided the local source of the poison be not discovered and effectually destroyed.

"Should infected ships, however, be allowed to remain at the wharves of a city until cold weather, they would undoubtedly so long continue to contaminate the air of their immediate vicinity, and consequently the disease would continue to occur, and perhaps, extend to a few of the adjacent streets. But such experiments are never permitted in the United States or in Europe. As soon as yellow fever shows itself, every thing suspected of containing foreign infection is removed, and of course any poison derived from this source is soon rendered innocuous by dispersion in the atmosphere." p. 82.

Thus then our author comes to the conclusion that the cause of real yellow fever—the Perkoino-miasma, may be imported into countries where it did not previously exist, and there spread endemically—at least, to a small extent. If this be the case with the *cause* of the disease, we do not see that it is very unreasonable to go a step farther, and allow that, under certain circumstances, the *fever itself* may be capable of generating its similitude. The following summary account of the manner in which *Perkoino miasma* appears and spreads through a city, as drawn from personal observation, we shall give in the words of its author.

"The grounds from which the miasm is exhaled, are usually of small extent, compared with the area over which it eventually spreads. At first, the poison is probably generated in a very minute quantity, perhaps not enough to occasion disease even in those who are the most susceptible to its noxious influence. But the quantity progressively increases, and shortly becomes sufficiently accumulated at and about its source to produce the few cases of fever which form the commencement of an epidemic. As the exhalation multiplies, it spreads to the adjoining streets, producing additional cases. At this period, however, the continuance of the disease as an epidemic, frequently appears doubtful, owing to the wind dispersing the miasm, the quantity of which is yet inconsiderable. But the poison, multiplying from day to day, slowly extends over a larger space, entering the houses, courts, and other retreats sheltered from the winds. As the season advances, the pestilential soil becomes more and more prolific of the poison, and when at length its exhalation is no longer increased, the epidemic soon rises to its height.

"In accounting for the extension of yellow fever, it is important to observe, that the quantity of *Perkoino miasma* daily augments, and that the principal cause of its not spreading rapidly with effect, is its dispersion in the atmosphere. The poison in a dilute state is, no doubt, always considerably in advance of the places in which it is sufficiently concentrated to produce disease; and although that portion of the miasm which is diffused through the streets of an infected district, may frequently be scattered by the wind so as to render them comparatively safe to passengers, yet as the poison has possession of enclosures and ranges of buildings, and is constantly emanating from its source, they soon become again pestilential in a calm state of the atmosphere. Moreover, it is probable that the miasm is condensed with the dews, and partially absorbed by the soil, from which it is exhaled during the heat of the day. This idea is the more plausible, seeing there is reason to believe that the specific gravity of *Perkoino miasma* is greater than that of atmospheric air, and that its elevation above the surface of the earth is never considerable. It is an old observation that the occupants of the upper stories of houses are less exposed to the ravages of pestilence than those who reside on the ground floors." 88.

Dr. Smith would fain identify the causes of plague and yellow fever; but evidently fears to urge this identity with any thing like confidence. He acknowledges that "the two disorders are distinguished by peculiar symptoms, and are somewhat different in their pathology," although they sometimes exhibit similar appearances. Again he grants that "yellow fever prevails in tropical countries, and never in the higher latitudes; whereas plague never appears within the tropics, but is confined to certain countries of the temperate zone, prevailing sometimes in northern countries where yellow fever is unknown." We really think it unnecessary to adduce stonger proofs of the dissimilarity of the two diseases and their causes than those which our author himself delivers in the foregoing passages.

Dr. Smith observes, that there are facts to prove that *perkoino miasma* is sometimes attached in such quantity to clothes, furniture, and other articles, that, on removing them from one place to another, as from ship to ship, or from town to the country, disease has thereby been communicated.

"With respect to yellow fever, such instances, though rare, explain the origin of some cases which are alleged to have arisen from contagion. Relying on the numerous statements relative to the more frequent communication of plague, in a pure atmosphere, we are inclined to believe that its poison is of *a grosser quality than that of yellow fever, and that it more strongly adheres to goods, and the persons and apparel of men.*" 90.

This poison is destroyed by the reduction of the atmospheric temperature to 32°, and in countries where frost never occurs, its production is suspended by rains, and a comparatively cool state of the air.

Genus II. Idio Miasma. *Species* 1. *Protidio Miasma.* This miasm is the ordinary source of genuine typhous fever. "It is produced from the excretions of the human body in unventilated houses, and in the apartments of the sick."

"Typhus from *Protidio miasma* is a disease of frequent occurrence in the cities of the United States. In the interior of the country, fevers arising from *this source* are comparatively rare; but the disorders originating from protokoino miasma, in their advanced stages, often resemble genuine typhus; indeed, the low or *typhoid* state of remittent fever, is doubtless occasioned in part by the morbid excretions of the patient reacting on his system. To this cause also may be partly ascribed the *typhoid* appearances, which are sometimes observed in atmospheric and contagious diseases, the nature of which is originally distinct from that of genuine typhus.

"The atmosphere in prisons, almshouses, and hospitals is extremely liable to contamination from *Protidio miasma*. The poison engendered in such places from crowding, uncleanliness, and want of ventilation, not only produces typhus, but has an agency in exciting the cachectic and strumous disorders which are so prevalent in humane and criminal institutions. These diseases are also frequent among the filthy poor, and are in some measure attributable to the same cause." 93.

*Species* 2. *Peridio-miasma.* Origin the same as the *protidio* or milder miasma, but elaborated under circumstances calculated to increase the virulence of human effluvia to its greatest malignity. These circumstances are usually the long confinement and crowding of individuals into apartments where cleanliness and ventilation are impossible, or totally neglected. Jail and ship-fevers sometimes originate from this deadly effluvium—witness the destructive consequences produced by *fomites* at the Black Assizes, and at the sessions of the Old Bailey, in 1750. *Peridio-miasmal* fever is synonimous with the typhus gravior of authors. The modern improvements in jails and hospitals have rendered this form of fever rare.

"The materials, however, which produce it, constantly exist in some places, in the form of protidio miasm, or human filth adhering to clothes, bedding, and furniture; and all that is necessary to convert those materials into the virulent poison in question, is their long confinement in a situation where the fresh air is excluded, and the temperature is favourable to chemical action." 97.

Our author has remarked before, that a combination of the terrestrial and human poisons may take place, forming an *idio-koino-miasma*, of various strength or virulence, and producing complications and varieties of fever, which we daily see, but which it is impossible to classify, or even describe. Under this head, and under the appellation of *peridio-koino-miasma*, (that is, a combination of human effluvium with the worst kind of terrestrial miasma) our author ranks the poison of plague and yellow fever. This he considers to be the cause of that pestilence which ravaged London in 1665, being called the poor's plague. The same was the agent in the plague of Athens.

*Remarks on the Species of Infection.*

"Having completed the arrangement of the infectious poisons into Genera and Species, we would now be distinctly understood as not intending to advance the opinion that each Species produces a disease as peculiar in its nature as Small-pox or Hooping-cough. The pathological phenomena which result from infection afford the strongest evidence that there is an affinity between its diseases. But this affinity has its limits. The dogma of the unity of disease derives no support from the similitude sometimes observed between different infectious fevers. Strictly speaking, a unity of disease can exist only where there is a unity of cause. If the same poison operate on individuals whose susceptibilities are different, grades of one disease will be the consequence. As a general truth, therefore, it may be said, that different poisons produce different disorders, each of which has different grades that collectively form an unit.

"It has long been a question whether yellow fever and plague are essentially different from intermittent and remittent fevers, or grades of the same disease. If our preceding views be correct, the two former must be regarded as specifically distinct from the latter; for yellow fever and plague are produced by the species *perkoino miasma*, while intermittent and remittent fevers arise from *Protokoino miasma*. These species and their varieties severally produce distinct fevers of various grades. This view of the subject is applicable to all the Species of Infection.

"The similarity of the different Species of infectious fevers depends upon the affinity of their poisons, which, as it was said before, are probably composed of the same elementary principles varied in their proportions. Now, so far as these poisons are allied to each other, so far only are the fevers occasioned by them grades of the same malady. Though there are phenomena which are common to all the miasmal diseases, yet there are others peculiar to each, which clearly indicate a specific difference in the poisons that produce them. In every febrile complaint there is an assemblage of symptoms which enables the experienced observer to ascertain its nature, and to discern its relations to other disorders.

"In the preceding classification, our object has been to exhibit, in a natural order, the various kinds of miasmata and their combinations, which are distinguishable in their origin and effects. To determine their physical and essential differences is impossible in the present state of science." 109.

The two orders of contagion and infection being now disposed of, we come to Dr. Smith's third order.

ORDER 3.—*Meteoration.* Under this head our author arranges all the atmospheric sources of disease, such as the vicissitudes of temperature and moisture, and those *occult influences* of the air which are occasionally experienced in every climate and season of the year, and which affect, in a peculiar manner, the animal and vegetable creation.* Our author properly observes that our knowledge of the unhealthy and diversified qualities of the atmosphere is entirely derived from the

* Dr. Smith excludes from this order all infectious and contagious effluvia. But when we find him admitting into it certain *occult influences*—we think he will be puzzled to account for *these* on any other principle than terrestrial effluvia. But more of this anon.

observation of their *effects*. "Thus, some disorders arise from the impressions of the *sensible* qualities of the air, while others arise from the influence of its insensible or occult properties." Upon these grounds he proposes to divide meteoration into two genera:—in the first will be comprised all those qualities which are manifest to the senses ("sensible meteoration")—in the second will be included all the insensible qualities of the air—"*epidemic meteoration.*"

Genus 1. *Sensible Meteoration.* This, as was said before, embraces the diurnal vicissitudes of the weather, and all the manifest qualities of the air, barometrical, hydrometrical, &c. The diseases produced by these are well known, as catarrh, croup, pneumonia, and other phlegmasial affections. The simple and direct effects of extreme atmospheric heat and cold are also to be included in this class. On this subject we need not dwell—indeed our author has merely referred to established works on atmospheric influence for information.

Genus 2.—*Epidemic Meteoration.* Here we are completely in the dark. Sydenham and Van Swieten, after watching the epidemic constitutions of many years in succession, came to the same conclusion—namely, that they did not thereby gain the least knowledge of the original causes of epidemic diseases! This sentiment has been re-echoed by all who have since written on the subject. The influenza—the cholera of India—the English sweating sickness, are notorious examples of epidemic meteoration. Our author has quoted the poet Armstrong in support of the *atmospheric* origin of these epidemics, and treats with entire scepticism the opinions of the older physicians, who supposed that epidemics were produced by "vapours exhaled from the bowels of the earth." Now as we have never been able to discover any change in the constitution of the atmosphere to account for endemic or epidemic diseases; whereas *miasmata from the earth* have been unequivocally proved to exist, (though their actual chemical qualities are unknown) have we not more grounds for believing that these *last* are the grand agents in epidemic disorders, than any occult change in the atmosphere itself, of which we have no proof whatever? Were the epidemic influence dependent on mere atmospheric change, how would a disorder travel progressively for hundreds of miles, directly against a regular trade wind, as was the case in India? Or how would an epidemic travel along one side of a river, destroying all before it, leaving the other side free, for many weeks—then, (the course of the wind being still the same) retrace its steps along the other bank, visiting the inhabitants with even-handed severity? Can it be possible to doubt that the cause of the disease was one of two things—either a miasma from the earth producing havoc as it was generated—or a specific contagion spreading along like that of small-pox or measles? The contagious character of these epidemics is now almost universally disbelieved —we have therefore only the other source to look to for the etiology of epidemics. Against these arguments we give our author all the support which may arise from his view of the etiology, as seen in the following passage.

"Allowing these observations to be well founded, the inquiry may be made, which of the imponderable elements is the most concerned in originating *Epidemic meteoration.* We know of no facts which shew that *light* has any agency in producing epidemics, and, as these diseases cannot be attributed to the vicissitudes of *temperature*, it appears that *caloric* is not, of itself, adequate to the effect. It seems probable, therefore, that *Epidemic meteoration* arises principally from variations in the quantity of electricity.

"If this view of the subject be correct, it is obvious, that many varieties of *Epidemic meteoration* may spring from certain quantities of electricity, concurring with certain proportions of the other variable constituents of the atmosphere; but in what manner these principles are combined with the other component parts of the air, or in what proportions they exist in the *varieties* of Epidemic meteoration, we shall not attempt to investigate: as yet, observation and experiment have made no progress towards elucidating this subject." 126.

And now, having brought Dr. Smith's etiological arrangement to a close, we shall present the reader with a synopsis of it at one view.

"*A Synopsis of the Remote Causes of Disease, as investigated and arranged in the preceding pages.*

"ORDER I.—CONTAGION.

"Genus I.—*Contagion communicable exclusively by contact.*

"*Species.*—Contagion of Itch, Syphilis, the Sibbens of Scotland, the Laanda of Africa, Frambæsia, or Yaws, Elephantiasis, or Leprosy, Hydrophobia, Vaccina.

"Genus II.—*Contagion communicable both by contact and by the Atmosphere.*

"*Species.*—Contagion of Small-pox, Measles, Chicken-pox, Scarlet Fever, Hooping-cough.

"ORDER II.—INFECTION.

"GENUS I.—*Koino Miasma.*

"*Species* 1.—Protokoino miasma—producing intermittent and remittent fevers.

"*Species* 2.—Perkoino miasma—producing yellow fever and plague.

"GENUS II.—*Idio Miasma.*

"*Species* 1.—Protidio miasma—producing the mild forms of typhus.

"*Species* 2.—Peridio miasma—producing the malignant forms of typhus.

"GENUS III.—*Idio-koino Miasma.*

"*Species* 1.—Protidio-koino miasma—producing the mild forms of compound fevers.

"*Species* 2.—Peridio-koino miasma—producing the malignant forms of compound fevers.

"ORDER III.—METEORATION.

"GENUS I.—*Sensible Meteoration.*

"Producing croup, pleurisy, and other phlegmasial disorders.—

"*Species*—undefined.

"GENUS II.—*Epidemic Meteoration.*

"Producing influenza, pneumonia typhoides, angina, and various other epidemic diseases.—

"*Species*—undefined." 129.

The second part of the work, embracing an "Inquiry into the Philosophy of Epidemics," we cannot at present enter upon—our design, in this article, being to draw the attention of the profession to some fixed and specific language and ideas on the subject of etiology. Although we have made objections to some parts of our author's arrangement, we think it is decidedly the best we have—indeed, we might say, it is the *only* etiological arrangement we possess—and, consequently, we recommend it to our brethren.

To Dr. Smith we return many thanks for the pleasure he has afforded us in the perusal of his work—which does great honour to transatlantic medicine.

